# Coping with the COVID-19 Pandemic in Italy and Spain: Lessons in Response Urgency

**DOI:** 10.7189/jogh.10.020326

**Published:** 2020-12

**Authors:** Pilar Montesó-Curto, Laura Sánchez-Montesó, Fabio Stefano Maramao, Loren Toussaint

**Affiliations:** 1 Faculty and Department of Nursing in Rovira i Virgili University, Campus Terres de l’Ebre, Tortosa, Spain; 2 Clinical and Research Resident, University of Rome Tor Vergata, Policlinico Tor Vergata, Rome, Italy; 3 Luther College, Decorah, Iowa, USA

##  “THE CONFINEMENT IN ITALY”

In Italy, on Friday, February 21, 2020, the first cases of infection were reported in the province of Lodi, 45 km from Milan. Codogno, a town of 15 000 inhabitants, placed the so-called “Patient 1” of Italy, a 38-year-old male, on the world COVID-19 map [1]. Until that moment the coronavirus was geographically distant. Within just 24 hours a “red zone” was established isolating 10 villages. A penal sanction of up to 12 years in prison was established for anyone who violated the isolation. In a little more than two weeks, the mayor of Codogno, Francesco Passarini, showed his satisfaction because there were no infections [2]. Nevertheless, on February 23, the Italian Prime Minister Giuseppe Conte signed a ministerial decree with the “confinement” measures for the regions of Lombardy and Veneto [2]_._ On Saturday, March 7, [3], a ministerial decree was approved by which the whole country was in a “red zone,” an area that was quarantined and had strict social distancing rules. A rumor about the decree having been leaked 3 or 4 hours before the official announcement allowed thousands of inhabitants of the affected area in the north of the country, especially Lombardy, to hastily return to their homes in other areas of the country with the risk of spreading the epidemic.

For Giuseppe Conte, Italy was experiencing the most serious crisis since the Second World War [1].

March 4, Italy closed all universities of all grades and majors and began teaching over the internet [3]. It took the Italian government just two weeks to react firmly with the laws enacted on March 7 to establish a “red zone” throughout the country, which is when it closed all shops, except pharmacies and food stores, and banned entry and exit from the country. On March 9, Italian citizens were banned from leaving their homes except for a justified reason. The Ministry issued a document to be carried by each citizen to justify any departures. On March 10 all but the most basic services such as hospitals, food stores, banks, kiosks and tobacconists were closed [3]_._

**Figure 1:**
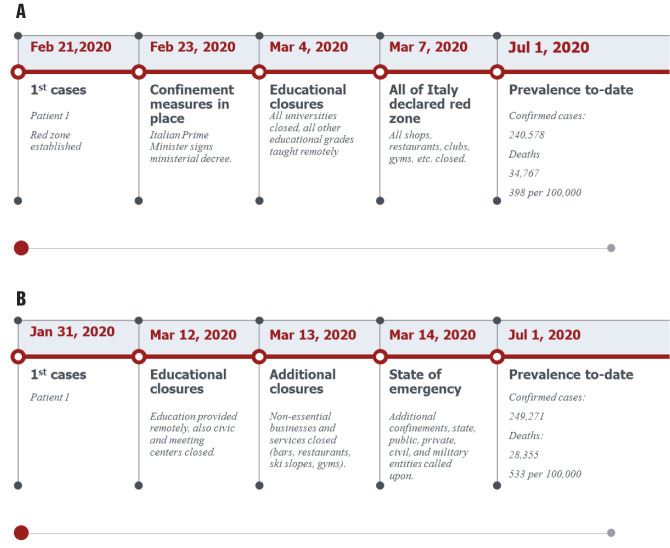
Italy’s (**Panel A**) and Spain’s (**Panel B**) response to COVID-19.

Indeed, Italy had already recorded 69 176 cases and 6820 deaths as of March 25, and it had become the European focus of the outbreak [4]. An overview of the timeline of events in Italy can be found in **Figure 1****,** Panel A.

## THE “STATE OF EMERGENCY” IN SPAIN

Spain documented its first case of COVID-19 on January 31, 2020. However, it wasn’t until March 12 that Spain took significant action. In fact, not quite one week prior, Madrid hosted the “8th International Women's Day” which Italian Professor Walter Ricciardi, a WHO executive and advisor to the Comte government, described as madness due to Italy's previous experience [1].

On March 12, the Catalan Government, chaired by Torra, ordered the closure of educational centers to stop the coronavirus epidemic. At the same time, the president of the Catalan Government asked for more measures, as those already taken seemed insufficient to him. At that time, 316 positive cases of COVID-19 had been reported. All educational centers were closed, including universities, where teaching would be provided virtually, as well as civic centers and meeting centers for retired people, and also toy libraries. On March 13, by order of the Catalan Ministry of Health, bars, restaurants, and all commercial spaces that are not necessities (ie, not dedicated to the sale of food) were ordered closed. Also the ski slopes, gyms, and entertainment venues such as discos were ordered closed. to contain the pandemic.

Then, on March 13, the Government of Catalonia decreed the “confinement” of several municipalities due to a cluster within the epidemic that caused an exponential increase in cases of COVID-19 in Igualada, Vilanova del Camí, Santa Margarida de Montbui i Òdena [5]. The departure of people who were not permanent residents in these affected municipalities was restricted. The March 14 decree arrived late, the measures were announced on Friday, March 13, they were approved on Saturday, March 14, so many citizens “escaped” in search of other homes and villages of origin.

A “state of emergency” in Spain was finally declared through a royal decree (463/2020) [6] on March 14 for a period of 15 calendar days. To carry out all the measures involved, it required a high degree of coordination in all its health policies, since 17 very diverse regions were included. The “State of Emergency” was declared by the Spanish Government appealing to the unity of the Spanish state affirming the harshness and difficulty that would be required to stop the spread of COVID-19. To this end, it announced the mobilization of “all the resources” of the State, public, private, civil, and military entities. Article 116.2 of the Constitution empowered the Government to apply this measure, using a decree agreed upon by the Council of Ministers, and for a maximum period of 15 days. On March 14, all face-to-face educational activities were suspended, leaving the home was allowed only for the purchase of basic necessities and work in activities declared essential.

For the approval of the extension of the “state of emergency”, the state needs the authorization of the Congress of Deputies. For this reason, on March 22, Sánchez met with the presidents of the Autonomous communities or regions by video conference. He was accompanied by the three ministers who formed the delegated authority in this crisis; the head of Health, Salvador Illa, the head of Defence, Margarita Robles, and the head of the Grande-Marlaska Interior and Transport and Mobility and Urban Agenda, José Luís Ábalos [7]. As of April 4, Spain was the second country in the world in infected people after the USA and the second in deaths after Italy with 11 744 deaths [1]. With a total of 809 deaths recorded on April 3, Spain recorded the lowest number of deaths per day for a week. The president of the government Sánchez called Pablo Casado, leader of the opposition, to inform him of his intention to extend “the state of emergency” from April 11_._ An overview of the timeline of events in Spain can be found in **Figure 1****,** Panel B.

**Figure:**
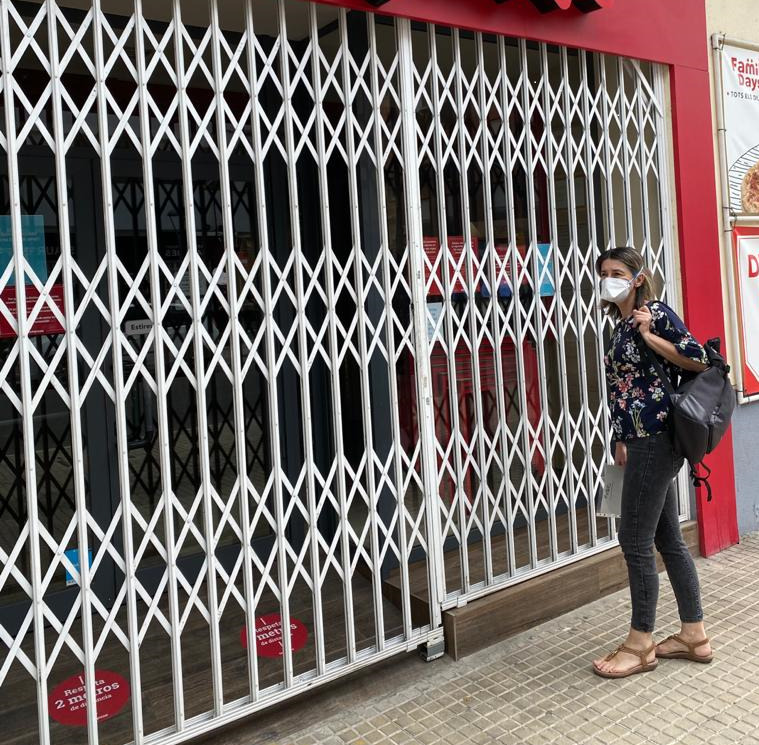
Photo: Closing of bars and restaurants in Catalonia. Source: (from the author's collection, used with permission).

## DIFFERENT RESPONSES IN SPAIN AND ITALY

Spain and Italy have had two different responses to the same crisis. The Spanish government’s response has been considered late by many [1]. In contrast to the direct, swift, and decisive action taken by the Italian government, the response in Spain was much more protracted. However, in the Catalan region of Spain the Catalan Government attempted to implement policies before the royal decree by the government of Spain chaired by Pedro Sánchez (463/2020) [6] to curb the spread of COVID-19. As of March 26, 2020, Spain had 56 188 cases of COVID-19, the fourth highest number in the world, and 4089 deaths, the second highest in the world. The Spanish Government held a plenary session in Congress on March 25 to address the COVID-19 crisis. The aim was to extend “the state of emergency” for an additional 15 days [8]. Meanwhile, Italy, which was ahead of Spain at the time of infection with COVID-19 and with the harshness of its policies in the face of the great impact of Northern Italy, proposed a “confinement.” This confinement period was longer than the 15 days of the quarantine ordered in Spain, until May 2 and then a progressive return to normal life little by little from May 16 [9].

The United States, which entered the COVID-19 pandemic later, claimed that New York was experiencing a “near-war atmosphere.” Also Trump recognized that “This is not a flu, this is ruthless. We are going to have a very hard two weeks. It is going to be painful, very painful for two weeks” [10]. Did the United States learn lessons about message framing and urgency of response? Perhaps, but were those lessons learned too late? The USA had the dubious distinction of leading the world in infections as of April 6, 2020, and continues to lead the world in cases at present.

Italy’s rhetoric was much more direct and discomforting and focused on “war” and that they must find the “ammunition,” that is, the resources to resolve it. In short, Italy used metaphors that reflect the danger detected and the necessary ways of managing the pandemic. Other countries should understand that the language that defines their policies is related to the actions that accompany them. Governments should be attentive to their rhetoric and metaphors and learn from previous experiences. Response urgency is crucial and should be reflected in government response and discourse.
